# A practical resource for multi-omics data integration in microbial systems

**DOI:** 10.1099/mgen.0.001860

**Published:** 2026-11-04

**Authors:** Warasinee Mujchariyakul, Abderrahman Hachani, Timothy P. Stinear, Kim-Anh Lê Cao, Benjamin P. Howden, Calum J. Walsh, Romain Guérillot

**Affiliations:** 1 Department of Microbiology and Immunology, The University of Melbourne at the Peter Doherty Institute for Infection and Immunity, Melbourne, Victoria, Australia; 2 Centre for Pathogen Genomics, The University of Melbourne, Melbourne, Victoria, Australia; 3 Melbourne Integrative Genomics, School of Mathematics and Statistics, University of Melbourne, Melbourne, Victoria, 3000, Australia; 4 Microbiological Diagnostic Unit Public Health Laboratory, Department of Microbiology and Immunology, Doherty Institute, University of Melbourne, Melbourne, Victoria, Australia

**Keywords:** biomarker discovery, microbial systems, multi-omics data integration

## Abstract

The increasing availability of microbial multi-omics datasets has created new opportunities to explore complex biological systems. However, exploration remains limited by the lack of accessible, reproducible workflows that integrate multiple omics layers and deliver easily interpretable visualizations of functional and pathway-level insights. Here, we present an R-based workflow for integrated analysis and network-based interpretation of microbial multi-omics data. The workflow enables microbiologists to analyse transcriptomic, proteomic and metabolomic datasets either individually or in combination, apply univariate and multivariate approaches for biomarker discovery and generate interpretable visualizations of functional and pathway-level signatures. Implemented as a multi-step R Markdown workflow, the resource leverages established open-source tools, including mixOmics for biomarker identification and omics integration and clusterProfiler for functional enrichment analysis. Functional integration is then performed on the enrichment results themselves rather than on individual features. By combining functional annotations from distinct omics layers, strains or conditions into a unified network, it enables simultaneous visualization of functional connectivity and of the sources from which each response was recovered, so that conserved and condition-specific responses can be identified directly rather than reconciled across separate outputs. A dedicated Shiny application makes this framework fully dynamic, allowing users to interactively filter, interpret and prioritize shared and condition-specific biological responses. We demonstrate its utility using a publicly available *Streptococcus pyogenes* dataset, revealing both shared and strain-specific functional responses to human serum. This resource provides an adaptable framework for systematic multi-omics analysis, improving accessibility and reproducibility and facilitating functional interpretation of microbial responses to diverse environments.

## Data Summary

The code for this workflow is available on GitHub (https://github.com/warasinee/Multiomics_Case_Study). Datasets from our previously published study [1] were used to showcase the functionality and practical utility of the workflow. The multi-omics *Streptococcus pyogenes* dataset used in this study is available in the following public repositories: Gene Expression Omnibus (GSE152821; GSE152822; GSE152823; GSE152824 and GSE152826), Proteomics Identifications Database (PXD020863) and MetaboLights (MTBLS2324) [1]. The corresponding data are provided in the Data folder of the GitHub repository. A dedicated Shiny app for interactive network visualization is available on the GitHub repository and at https://warasinee.shinyapps.io/Network_App.

**Impact Statement**

High-throughput omics technologies are transforming our understanding of how microbes adapt to diverse environments and cause disease. The integration of diverse omics layers at a systems level, combining transcriptomics, proteomics and metabolomics data to identify signature molecules, pathways and their interactions, remains challenging. Here, we present an R-based bioinformatic workflow designed for microbiology research, which connects existing tools and customized functions to streamline data integration and interpretation. The workflow links biomarkers to functional pathways, visualizes results in an interactive network context and is designed for flexibility and reproducibility. This practical resource lowers technical barriers to microbial multi-omics analysis, providing user-friendly access for dataset exploration and integration and supporting interpretation of systems-level microbial adaptation in environmental, clinical and industrial contexts.

## Introduction

Advances in high-throughput technologies have enabled microbiologists to generate diverse omics datasets, capturing gene expression, protein abundance and metabolite levels, within the same system. Combined, these data offer a multi-layered view of microbial physiology and adaptation, providing opportunities to uncover coordinated responses that isolated single-omics analysis cannot capture. This systems-level perspective contrasts with the traditional reductionist approach often used in molecular microbiology [2], which dissects complex systems into individual components and examines their interactions [3]. While reductionism remains essential for elucidating discrete molecular mechanisms, it is insufficient for the understanding of phenotypic traits displayed by whole systems. Consequently, multi-omics integration is emerging as a cornerstone of systems microbiology, with applications spanning from bacterial metabolism and stress responses to host–microbe interactions and microbiome ecology [1, 4, 5].

Despite the promise of multi-omics, analysing these datasets remains challenging. Current workflows usually employ one of two approaches. In early integration, different omics layers (i.e. genes, proteins and metabolites) are analysed together from the outset using multivariate methods. A widely used example is DIABLO (Data Integration Analysis for Biomarker discovery using Latent variable approaches for Omics studies), which implements supervised multiblock partial least squares discriminant analysis (PLS-DA) to uncover correlated features across datasets in relation to experimental conditions [6]. This strategy helps reveal coordinated biological signals across omics layers. In contrast, late integration involves analysing each dataset separately and then combining the results afterwards, often through pathway enrichment approaches. Two common enrichment methods are over-representation analysis (ORA), which tests whether certain pathways contain more differentially expressed genes than expected by chance, and Gene Set Enrichment Analysis (GSEA), which evaluates whether predefined sets of genes are enriched at the top or bottom of a ranked list of all genes [7]. As both strategies are complementary, their integration within a single framework offers a more comprehensive view. To further refine biological interpretation, a list of genes can be contextualized through network-based approaches, including gene regulatory networks, co-expression networks, protein–protein interaction networks and metabolic networks [8–10]. These networks are often presented as graph-structured data that incorporates prior knowledge. In these representations, entities are presented as nodes, and their relationships are modelled as edges. Existing biological knowledge graph frameworks, such as BioCypher and MetalinksDB, have largely focused on human-centric biomedical applications [11, 12].

Here, we present an R Markdown-based workflow that integrates established packages (mixOmics and clusterProfiler) with custom code and functions to connect outputs across different analytical stages. The workflow integrates at the junction between signature discovery and functional enrichment: functional integration is performed on the enrichment results themselves rather than on individual features. It supports both univariate and multivariate analyses and accepts inputs from either early or late integration. By situating biomarkers within functional networks, the workflow enables microbiologists to explore conserved and condition-specific responses across strains, omics layers and experimental conditions (Fig. 1). Here, condition-specificity information (e.g. conserved and strain-specific responses) is overlaid onto network structure, enabling enhanced visualization and comparative systems-level analysis. Integrating at this level is what makes heterogeneous sources comparable. Transcripts, proteins and metabolites occupy different identifier spaces. Bacterial isolates of the same species differ in accessory gene content, and analogous functions are frequently encoded by non-orthologous genes, so feature-level comparison registers such cases as missing data even when the same function is performed in both strains. Component loadings (multivariate models), binary feature selections (sparse variants of those models) and log fold changes (logFCs) [differential expression (DE)] are likewise not directly comparable quantities. Converting the outputs of any of these analyses into enriched functional annotations provides a shared ontology in all three cases, so results from different omics layers, strains and analytical strategies can be integrated. Because the functional integration acts downstream of the analysis, the analytical strategy can be chosen to suit the biological question, or several applied in parallel, without changing the integration step.

**Fig. 1.**
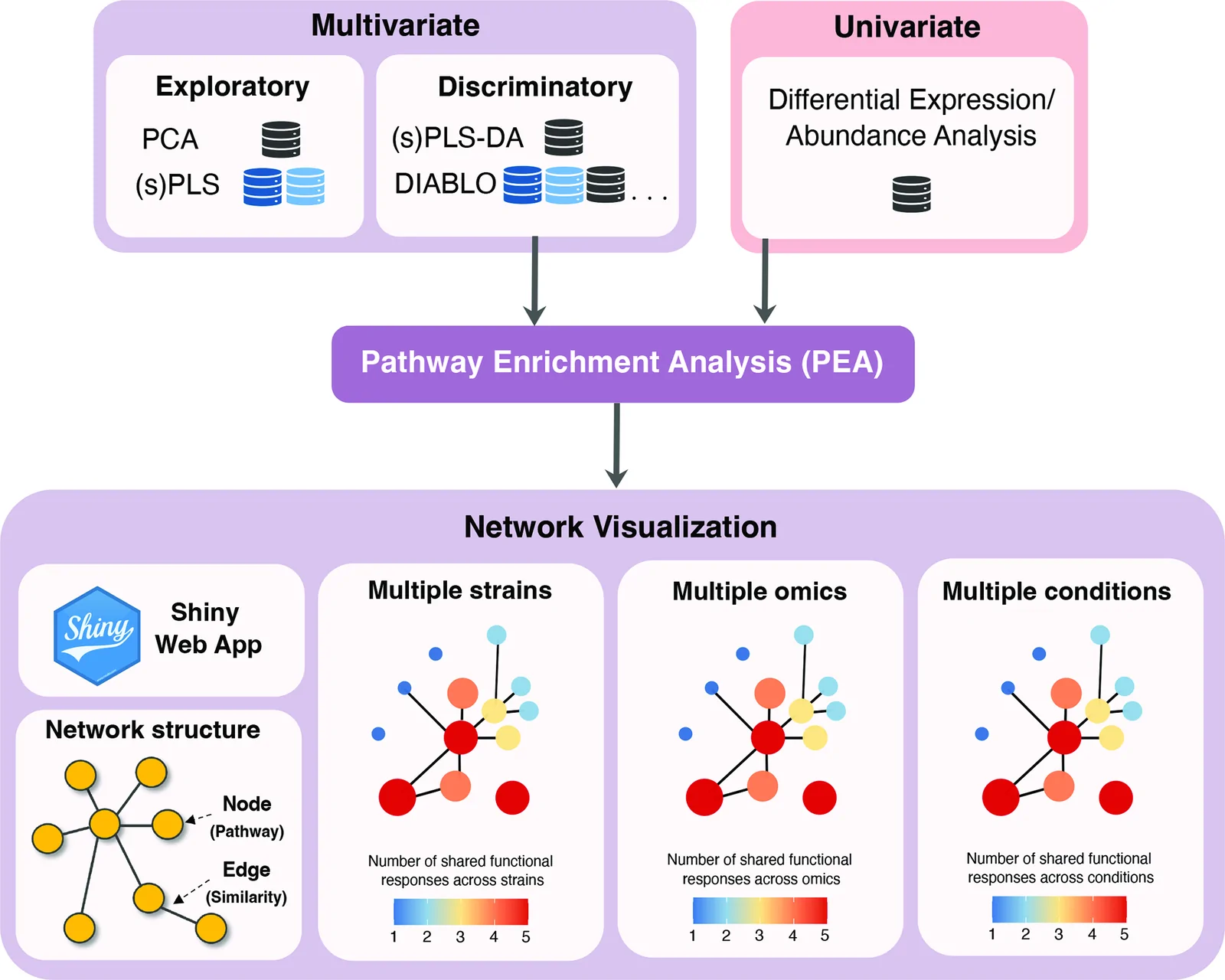
Network integration of functional enrichment results. Signature molecular features identified from multivariate (i.e. (s)PLS-DA and DIABLO) and univariate (i.e. DE analysis) approaches are used as inputs for functional/Pathway Enrichment Analysis (PEA). Integration is performed on these functional annotations rather than on the features themselves, with nodes representing pathways or other significantly enriched functional annotations and edges connecting functionally related pathways and functions. Node size and colour indicate the number of strains, omics layers or conditions in which each function was recovered, and edge width indicates the number sharing a given relationship or pathway similarity, so that functional connectivity and condition specificity are shown together. This enables the identification of shared and condition-specific functional responses across different strains, omics layers or multiple tested conditions. The core elements of the workflow are highlighted in purple. A Shiny app was developed to allow users to interactively view, filter and prioritize the results of the integration of functional enrichments. PCA, Principal Component Analysis; (s)PLS-DA, (sparse) PLS-DA.

The resource is aimed at microbiologists without formal bioinformatics training, for whom the barrier to multi-omics analysis is the absence of a fully documented route from processed omics data to functional interpretation. It is therefore presented as an annotated R Markdown covering several use cases and integration strategies, so that each analytical decision remains visible and modifiable rather than concealed within an analytical black box. This resource is designed to lower technical barriers, enhance reproducibility and support the functional interpretation of multi-omics data, helping microbiologists translate complex datasets into biological insight across diverse microbial systems.

## Existing methods and their integration in the workflow

Traditional DE analysis remains a cornerstone of omics research, identifying individual genes or molecules that vary between conditions. While powerful, this univariate approach considers each feature in isolation and may overlook coordinated patterns. Multivariate methods, such as those implemented in the mixOmics framework [6, 13], address this limitation by analysing many features simultaneously. Techniques like principal component analysis (PCA) allow researchers to explore overall data structure, while (sparse) PLS [(s)PLS] and PLS-DA highlight sets of molecules that best discriminate between biological conditions. In multi-omics integration, multiblock (s)PLS-DA (i.e. DIABLO) extends these methods to uncover signatures that are consistent across different omic layers, providing a systems-level view of bacterial responses [6, 13]. Importantly, univariate and multivariate analyses often yield distinct yet complementary insights that together help explain complex biological systems. The signature molecules identified through these statistical approaches can then be combined with prior knowledge of underlying molecular pathways and functional annotations using pathway enrichment analysis (PEA) such as ORA and GSEA. To further aid interpretation, this workflow uses the concept of a pathway similarity network to construct networks based on shared molecular components [14, 15]. In such a network, if two pathways are related, the two corresponding nodes are connected by an edge.

Here, our pathway integration approach highlights conserved functional responses and supports application to a wide range of multi-omics study designs and biological questions. This network-based integration of functional enrichment can be used to compare responses across bacterial strains or to combine results from different omics layers and multiple tested conditions when focusing on a single strain (Fig. 1). The workflow facilitates the identification of biomarkers and signature molecules, contextualizing them within pathways and functional interactions to support hypothesis generation and guide functional validation. We have also developed a web-hosted Shiny app for interactive viewing, filtering and prioritization of functional enrichment results.

This workflow leverages publicly available R packages and commonly used software, supplemented with custom R functions to connect different analyses’ outputs. The workflow is implemented in R Markdown documents that interleave executable code with explanatory text, creating an annotated, step-by-step guide for visualizing and interpreting omics results. The workflow supports two main analyses (Fig. 2):

**Fig. 2.**
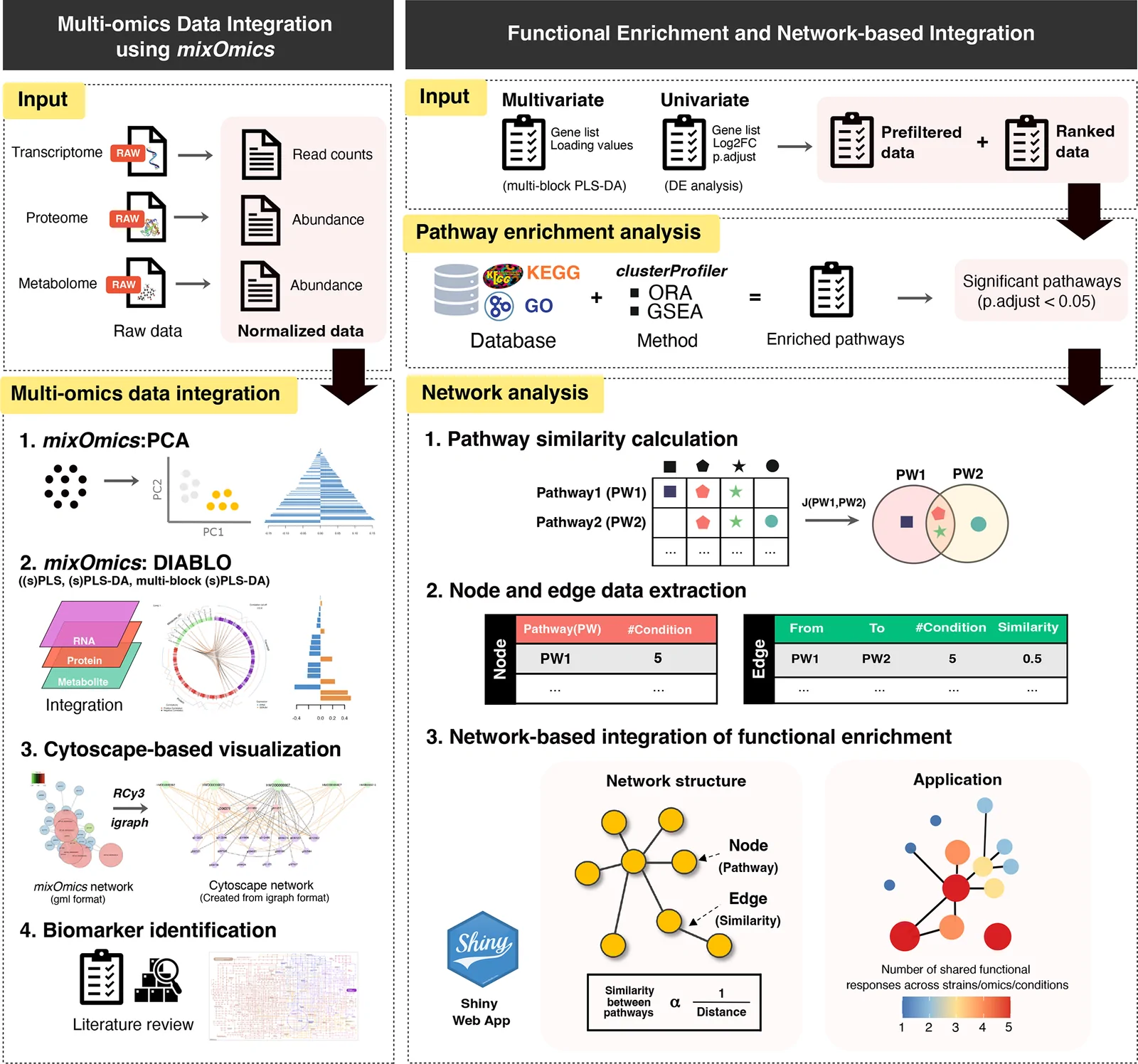
Analytical frameworks for multi-omics data integration and network integration of functional enrichment results. The workflow supports multi-omics data integration using mixOmics, including dimensionality reduction (PCA) and supervised integration (DIABLO) to identify key biomarkers and functional relationships across omics layers. Correlations of significant biomarkers across omics layers can be visualized directly in R or in Cytoscape [43], which can be controlled from R using the RCy3 package [44] (left). It also supports functional enrichment and network-based integration to enable interpretation of enriched pathways across conditions (right). Fig. S1 (available in the online Supplementary Material) and Supplementary Methods contain a more detailed computational workflow to support user analytical decision-making and parameter tuning of multivariate models.

1. **Multi-omics data integration using mixOmics**
  - **Dimensionality reduction and correlation analysis** using PCA and PLS methods in mixOmics, enabling exploration of global data structure and relationships across omics layers.
  - **Supervised integration and classification** with DIABLO and sPLS-DA from mixOmics [6, 13], which uncover correlated features across datasets and highlight discriminative signatures linked to experimental conditions.

1. **Functional enrichment and network-based integration.**
  - **PEA** through clusterProfiler, supporting both ORA and GSEA [7], and allowing use of inputs from both univariate DE results and multivariate feature rankings.
  - **Network-based integration of functional enrichment** of enriched pathways using interactive visualization through a dedicated simple Shiny app.

Together, these tools situate biomarkers within functional networks, revealing conserved and condition-specific responses across strains, omics layers and experimental conditions. The workflow thus lowers technical barriers, enhances reproducibility and supports hypothesis generation by linking statistical outputs directly to biological interpretation.

## Interactive network visualization via a Shiny web application

Complex biological data can be presented as networks, in which nodes and edges represent entities and their relationships, respectively. In this workflow, networks are constructed based on shared molecular components. Here, nodes represent pathways or other significantly enriched functional annotations and edges connect functionally related pathways. To support interactive exploration of enrichment pathway networks, we developed a dedicated R Shiny web application called Network of Enriched Pathways (https://warasinee.shinyapps.io/Network_App/). The application provides a graphical interface for filtering, visualizing and exporting the network. It requires two tab-separated input files (node and edge tables generated by the workflow; Fig. 1). The control panel is divided into six subpanels in a sidebar layout: upload data, network filter, visualization style, interactive plot, static plot and node ranking (Fig. 3). To generate the initial plot, after uploading the node and edge tables or selecting example datasets (*Staphylococcus aureus* and *Streptococcus pyogenes*), users can specify a condition threshold to restrict which nodes are displayed in the network filter panel. The number of conditions displayed can be modified to accommodate both specific and broad comparisons. For further node modification, users can select the colour palette or define custom hexadecimal codes and select legend labels (strains, conditions or omics) to reflect the experimental context in the visualization style panel. The interactive plot supports real-time exploration through zooming, panning and node dragging. Four network layouts are available: Force-directed (Fruchterman–Reingold), Kamada–Kawai, Circular and Hierarchical. Node size and colour encode the number of conditions shared in each pathway. Edge thickness is proportional to the Jaccard similarity between connected pathway pairs or the number of conditions sharing the same interaction. Clicking on any node displays a tooltip showing the pathway name, condition count and the list of condition identifiers, facilitating rapid identification of shared versus condition-specific enriched pathways. Nodes can be excluded by name to remove uninformative terms, and a similarity-based grouping function allows the merging of highly similar pathways into a single node, reducing visual clutter from a dense network. A node ranking table below the interactive plot summarizes each pathway by its number of conditions and network degree (interactions) and allows sorting in ascending or descending order. The static plot supports publication-ready figures, allowing export as a portable network graphic (PNG) or a scalable vector graphic (SVG) for further editing.

**Fig. 3.**
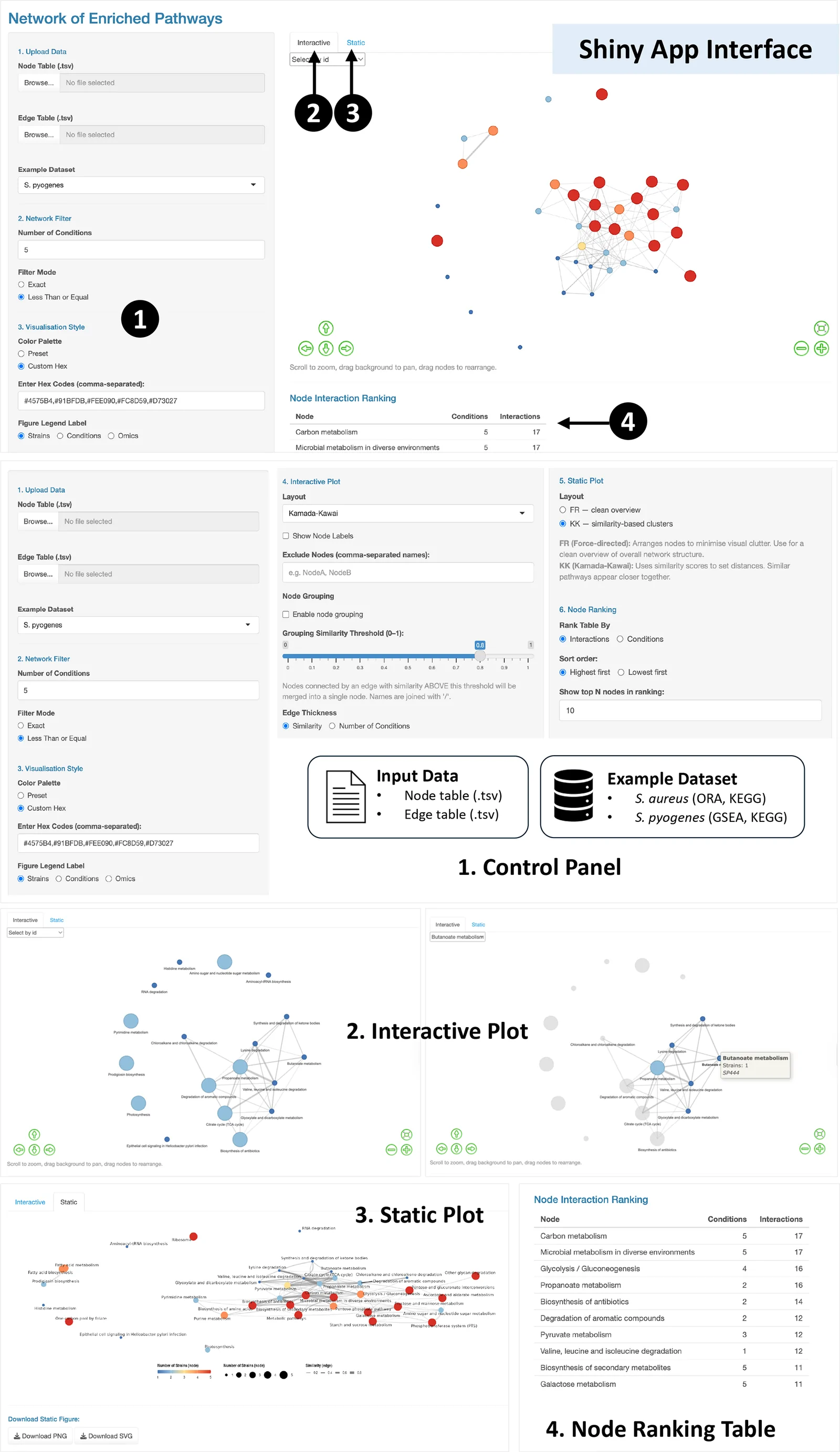
Structure of a Shiny web application for interactive network visualization. There are four main parts in this application: (1) control panel, (2) interactive plot, (3) statistic plot and (4) node ranking table.

The app includes a ranking and filtering system allowing prioritization of the most informative and discriminatory pathways across conditions. The ‘network filter’ enables users to select pathways based on how many conditions they appear in. This supports direct isolation of pathways that are conserved across all conditions (core or conserved responses) or unique to a single condition (strain-specific or treatment-specific responses). The ‘node ranking’ table ranks pathways by either the number of conditions they appear in or the number of interactions they have. A node with a high condition count and many interactions indicates a broadly conserved, highly connected pathway (e.g. core metabolic response). A node with a low condition count and few interactions indicates a narrow, isolated pathway (e.g. condition-specific pathway). The node prioritization directly facilitates biomarker identification. By combining the network visualization with the ranking table, users can short-list candidate pathways for further studies.

## Preprocessing, functional annotation and data inputs for the workflow

Preprocessing and quality control are essential prerequisites that lie outside the workflow but ensure reliable downstream analysis. For transcriptomic data, this typically involves read quality assessment, trimming, alignment and normalization of counts to correct sequencing biases. Proteomic datasets require spectral QC, peptide identification with false discovery rate control and normalization of quantification values to reduce technical variability. Metabolomic data undergo peak detection and peak alignment across samples, outlier filtering, imputation of missing values and normalization against internal standards to account for instrument drift. Collectively, these steps minimize noise and remove low-quality signals to enhance the biological relevance of inputs carried forward into downstream multi-omics integration and functional analysis. For RNA-seq data, *DESeq2* [16], *edgeR* [17, 18] and *limma-voom* [19] are statistical R packages used for normalization and identification of differentially expressed genes from sequence read counts, with *edgeR* and *limma-voom* implemented in the easy-to-use web tool *degust* [20]. *MetaboAnalyst* [21], *MZmine 3* [22] and *XCMS* [23] are popular processing pipelines from raw MS data.

Variability introduced by different preprocessing pipelines can significantly impact downstream integration, as multivariate methods such as DIABLO and PLS-DA are highly sensitive to systematic variations or batch effects [6, 13]. Such variations are strong confounders in the statistical analysis, potentially preventing the identification of true biological signals [6], introducing biases towards specific omics types [13], introducing value instability, thus rejecting biologically relevant markers [24], increasing the risks of data overfitting [13] and clustering inaccuracy [24].

Functional gene annotation is essential for linking features to biological pathways and categories. For functional mapping, dedicated microbial annotation pipelines such as Prokka [25], Bakta [26], BASys2 [27], MicrobeAnnotator [28] and eggNOG-mapper [29] are recommended, as they provide per-feature assignments to Kyoto Encyclopedia of Genes and Genomes (KEGG) Orthology (KO) groups, Gene Ontology (GO) terms, evolutionary genealogy of genes: Non-supervised Orthologous Groups (eggNOG), or Clusters of Orthologous Groups (COG), enabling consistent functional annotation across datasets. The workflow requires three main inputs: (i) normalized abundance matrices (features×samples) for each omics layer; (ii) DE results table containing identifiers, experimental metadata and statistical outputs (e.g. logFC and False Discovery Rate (FDR)-adjusted p-value); and (iii) functional annotation mappings that link features to pathways or functional terms. Detailed specifications of input file formats, recommended preprocessing steps and example datasets are provided in the GitHub repository to ensure reproducibility and compatibility with the workflow.

The described workflow focuses on the integration and functional interpretation of multi-omics data. Therefore, we treat missing-value imputation, batch-effect correction and platform-specific normalization as essential prerequisites that are performed within preprocessing pipelines, prior to data loading into the workflow. To ensure the reliability of downstream analysis, we recommend following the preprocessing standards for each omics dataset type [30–32] or using the recommended steps on an established platform like Omics Playground [33]. While many multivariate methods require complete matrices, the mixOmics framework is specially designed to handle missing data using the non-linear iterative PLS (NIPALS) algorithm, which estimates latent components even when some data points are missing [13]. Because DIABLO utilizes a consensus voting scheme (majority or weighted vote), it can still predict the group of a sample even in the absence of one or more omics blocks [6]. In other words, class prediction of the DIABLO framework relies on individual votes from each omics dataset, which allows for some missing datasets during the prediction step.

## Case study: Multi-omic characterization of *Streptococcus pyogenes* response to human serum

We demonstrate the workflow on a published multi-omic dataset for *S. pyogenes* comprising transcriptomic, proteomic and metabolomic data [1]. In this dataset, samples were collected from 5 clinically relevant strains grown under 2 conditions – Roswell Park Memorial Institute (RPMI) or human serum – with 6 biological replicates per condition, yielding a total of 60 samples. Details of the computational resources, data formats and software dependencies in this case study are provided in Supplementary Methods and Tables S2–3.

## Unsupervised single-omic analysis

To assess the similarity of bacterial responses to human serum, we performed PCA across all omics datasets. The primary source of variation corresponded to the growth condition, indicating distinct global profiles between serum- and RPMI-grown bacteria (Fig. 4a). Each dataset showed clear separation between conditions, suggesting that *S. pyogenes* undergoes a substantial adaptation to human serum. Shared (conserved) responses across strains accounted for most of the variation, whereas strain-specific effects were comparatively minor (Fig. 4b). Notably, the transcriptomic and metabolomic profiles of strain SP444 differed from those of other strains, while the proteomic data showed weak strain-related separation along the first two principal components (Fig. 4b and c). To further explore relationships between omics layers, we applied Projection to Latent Structures, also known as PLS. This approach models the covariance between datasets to assess whether shared information exists across layers. Pairwise PLS analyses revealed strong correlations (*r*>0.9) between the first latent components of the omics datasets, highlighting a high degree of co-variation and, therefore, a coordinated biological response among the omic layers.

**Fig. 4.**
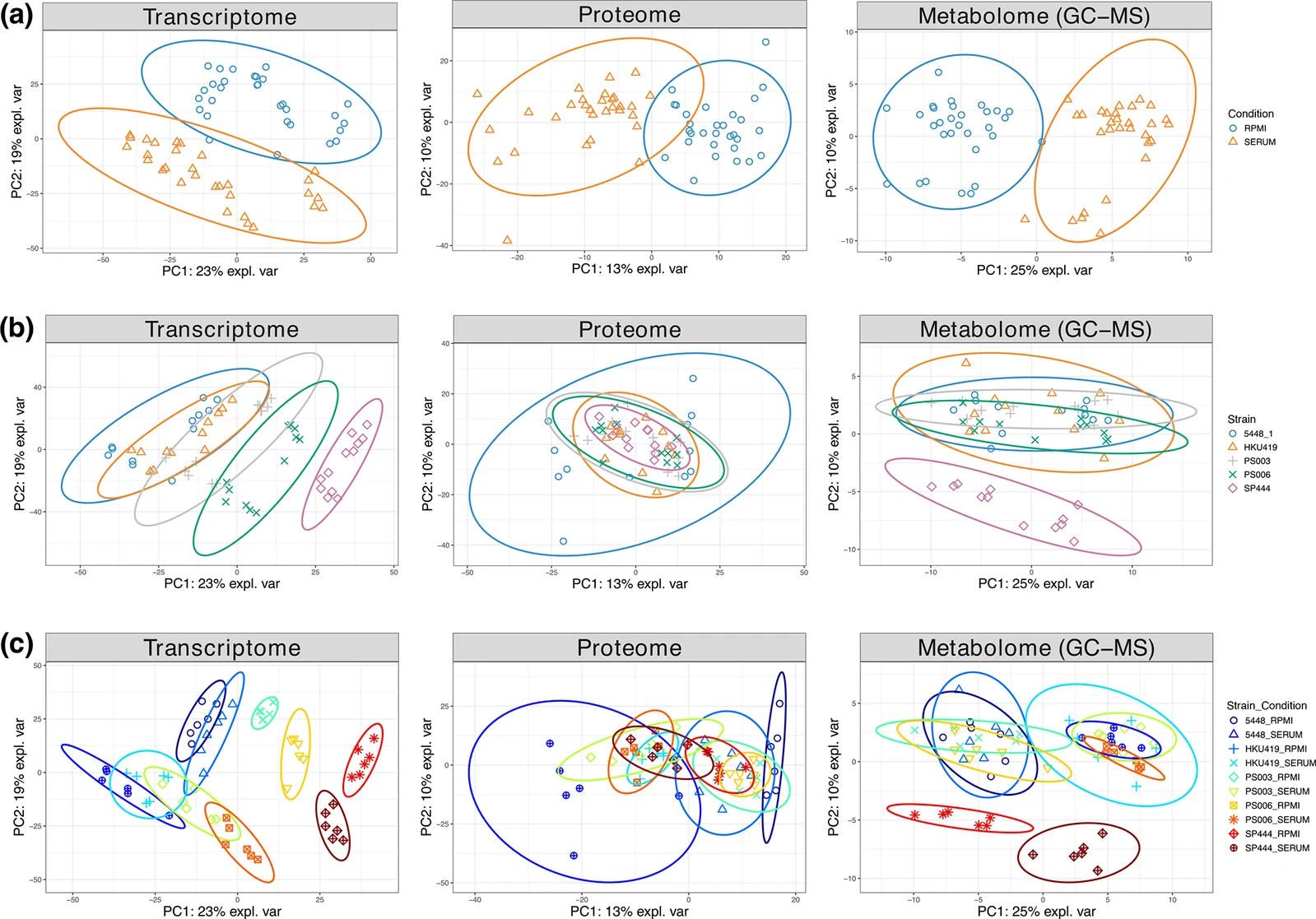
Unsupervised, single-omic analysis of *S. pyogenes* response to human serum using unsupervised PCA. PCA plot illustrating the overall similarity between samples across transcriptomic, proteomic and metabolomic (GC-MS) datasets. Samples are coloured by growth condition (**a**) or strain (**b**) or a combination of strain and condition (**c**).

## Supervised single-omic analysis

Next, we applied a supervised classification approach using the sPLS-DA framework to identify features that best discriminate between serum and culture media conditions. This analysis demonstrated the enhanced separation power of the supervised method compared to the unsupervised PCA (Fig. 4a and b), particularly for the proteomic dataset (Fig. 5a and b), where clustering by condition and strain becomes more distinct. Samples separated by media condition along the first component, while strain-specific variation was primarily captured by the second component.

**Fig. 5.**
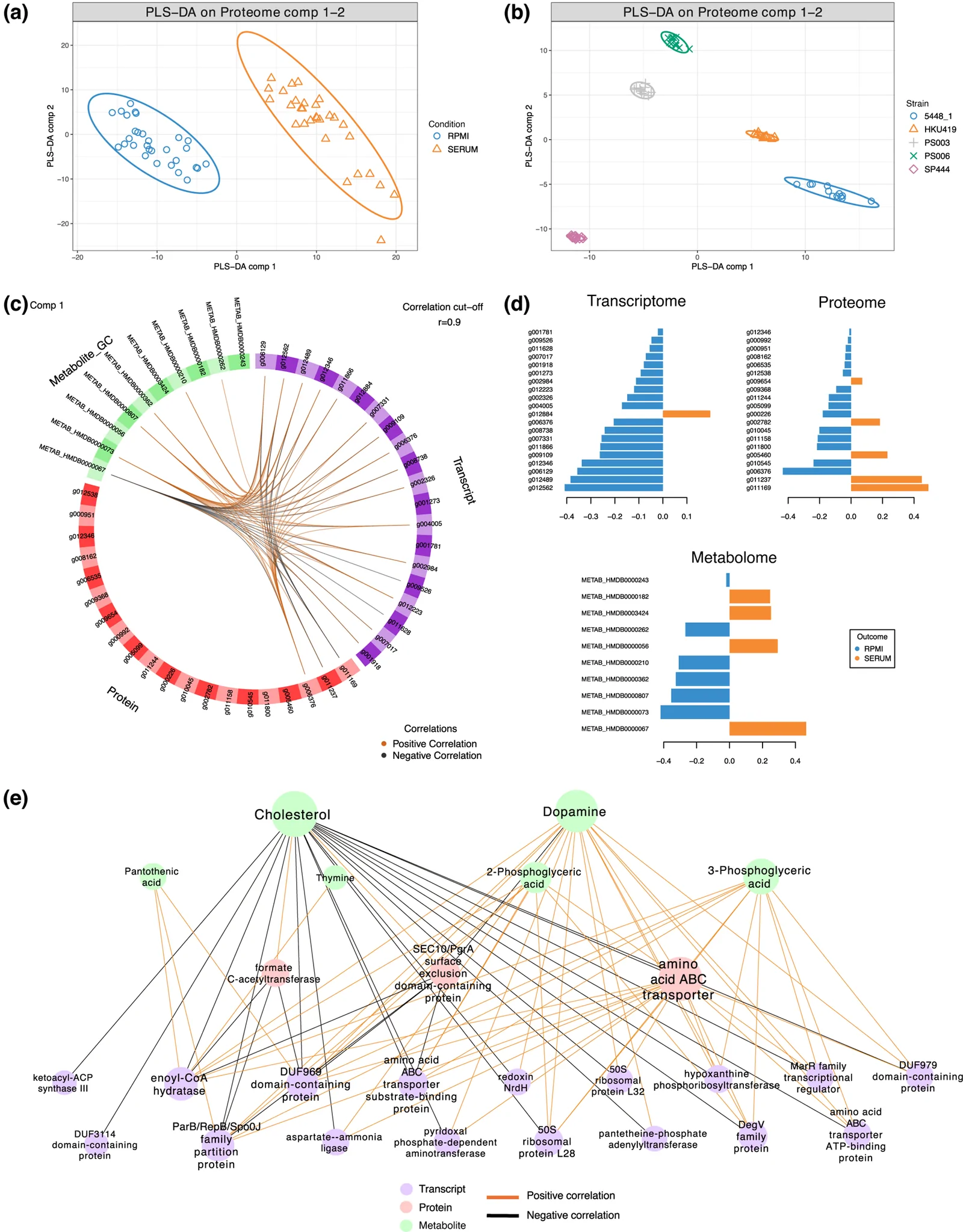
Supervised single- and multi-omic analysis of *S. pyogenes* response to human serum. (**a–b**) Supervised classification using the sPLS-DA framework. Sample plots from sPLS-DA on the proteomic dataset show discrimination of samples by media condition (**a**) and strain (**b**). (**c–d**) Supervised multiblock sPLS-DA (DIABLO) integrating transcriptomic, proteomic and metabolomic datasets. (**c**) Circos plot showing pairwise correlations (*r*>0.9) between features across omics layers. (**d**) Loading plots illustrating the most discriminatory features along the first component for each dataset. (**e**) Network visualization of correlated features, adapted from (**c**). Edges represent correlations, with edge colours indicating positive (orange) or negative (black) correlations. Node sizes indicate the number of interactions, and node colours represent data type: purple, transcripts; pink, proteins; green, metabolites.

## Supervised multi-omic analysis

We then employed multiblock sPLS-DA (DIABLO) to integrate multiple omics datasets in a supervised framework. The model was trained using labelled data corresponding to the two conditions (RPMI and human serum) and identified highly correlated features across omics layers that maximally discriminate between these conditions (Table S1; Fig. 5c–e). Due to the high correlation across all omics datasets (pairwise PLS comparisons, *r*>0.9), the initial model was generated using a design matrix of 0.5 to balance class separation and cross-omics correlation structure (see Supplementary Methods for a detailed explanation of the design matrix). The number of components was selected based on model performance during cross-validation, where improvements in balanced error rate (BER) are assessed to determine whether additional components provide meaningful gains. In our case study with small sample sizes, leave-one-out cross-validation was used in the parameter tuning step of the DIABLO model. The result suggested that using only the first component achieved the lowest BER (overall BER=0). Feature selection is performed simultaneously, where the optimal number of variables per layer is defined as the subset size achieving the lowest BER during tuning. Feature selection was performed using the *tune.block.splsda* function, which evaluated ~250 candidate models using repeated cross-validation across a predefined grid of feature numbers (ranging from 10 to 100 per omics block). The optimal configuration identified retained feature sets of (20, 20 and 10) across the three layers, corresponding to a total of 50 features. These selected features represent the most discriminative and highly correlated molecular signatures across the multi-omics dataset, as determined by minimization of the BER. The final model performance was further evaluated using Receiver Operating Characteristic (ROC)-based metrics to assess classification performance. The loading values derived from the DIABLO model (Table S1; Fig. 5d) reflect the importance of each feature in driving this separation along the first component. Features with larger absolute loading values are the key contributors to the observed differences between conditions. The direction (>0 and <0) of these loadings further indicates condition-specific responses – positive loadings correspond to serum-enriched responses, while negative loadings correspond to RPMI-associated features.

In 2023, Mu and team demonstrated, using a late integration approach, that (i) a high abundance of host-derived cholesterol in *S. pyogenes* exposed to human serum is associated with functional adaptation rather than serving as a nutrient source and (ii) cholesterol bound to bacteria significantly increased their resistance to the human antimicrobial peptide LL-37 [1]. Applying our supervised multi-omics workflow to the same *S. pyogenes* multi-omics dataset, we identified cholesterol as a key biomarker of serum responses. Our model revealed a strong negative correlation between cholesterol levels (metabolomics) and the expression of genes associated with the FASII pathway (transcriptomics; Ketoacyl-ACP synthase III and Enoyl-CoA hydratase), indicating that *S. pyogenes* modifies its metabolism to process host-derived cholesterol. We also identified a negative correlation with purine metabolism. This finding is consistent with the work of Lambert and colleagues [34], who reported that disrupting FASII repression led to the upregulation of the purine synthesis operon and decreased fitness during infection. Additionally, another study has shown that deletion of the FASII pathway repressor FabT in *S. pyogenes* significantly reduced bacterial survival in human whole blood [35]. Together, these highlight the importance of streptococcal fatty acid biosynthesis during exposure to host blood. By employing early integration (DIABLO), our workflow successfully identified coordinated multi-omics signatures and discovered biological relationships important for *S. pyogenes* pathogenicity and survival.

## Pathway Enrichment Analysis

Building on the discriminant and highly correlated variables identified by the DIABLO model, we performed PEA to explore the biological context of these cross-omic associations. All pathway enrichment results (ORA and GSEA) were generated via the clusterProfiler package. In this case study, an adjusted *P*-value (from FDR correction using the Benjamini–Hochberg method) threshold of <0.05 was used for statistical significance, whereas a *P*-value threshold of <0.05 was used for exploratory purposes. This analysis revealed that carbon, lipid and nt metabolisms, along with defence mechanisms, were strongly elicited in *S. pyogenes* in response to serum (Table S4).

To explore the functional relationships among enriched pathways, we used network-based integration of pathway enrichment results using Jaccard similarity scores to quantify gene overlap between pathways. This approach moves beyond static pathway lists by visualizing functional connectivity, revealing both shared (conserved) and strain- or condition-specific responses. Pathway-level networks can be generated from three complementary analyses: (i) enrichment of discriminant features identified by multi-block PLS-DA, highlighting shared serum responses across strains (Fig. S2A); (ii) pathway enrichment from single-omics DE analyses, showing both shared and strain-specific responses (Fig. S2B); and (iii) integration of responses across multiple omics layers within a single strain (SP444) (Fig. S2C). Here, we demonstrated the application of the Shiny app (https://warasinee.shinyapps.io/Network_App/) to visualize the network of enriched pathways using a *S. pyogenes* transcriptomic dataset as an example (Fig. 6a). Pathway enrichment was derived from GSEA against the KEGG database, with statistical significance defined as an adjusted *P*-value<0.05. In addition, network filtering was applied to enhance visualization and interpretation (Fig. 6b). In this case, we filtered out broad categories (e.g. metabolic pathways, carbon metabolism, microbial metabolism in diverse environments, biosynthesis of antibiotics, biosynthesis of secondary metabolites and biosynthesis of aas) and performed node grouping (grouping similarity threshold=0.8) to merge highly similar pathways into a single node (e.g. fatty acid biosynthesis/fatty acid metabolism). The interactive plot supports real-time exploration, allows researchers to identify and prioritize key pathway functions, and provides a foundation for generating testable hypotheses. Importantly, clicking on any node displays a list of strain identifiers sharing enriched pathways, facilitating rapid identification of conserved and strain-specific responses.

**Fig. 6.**
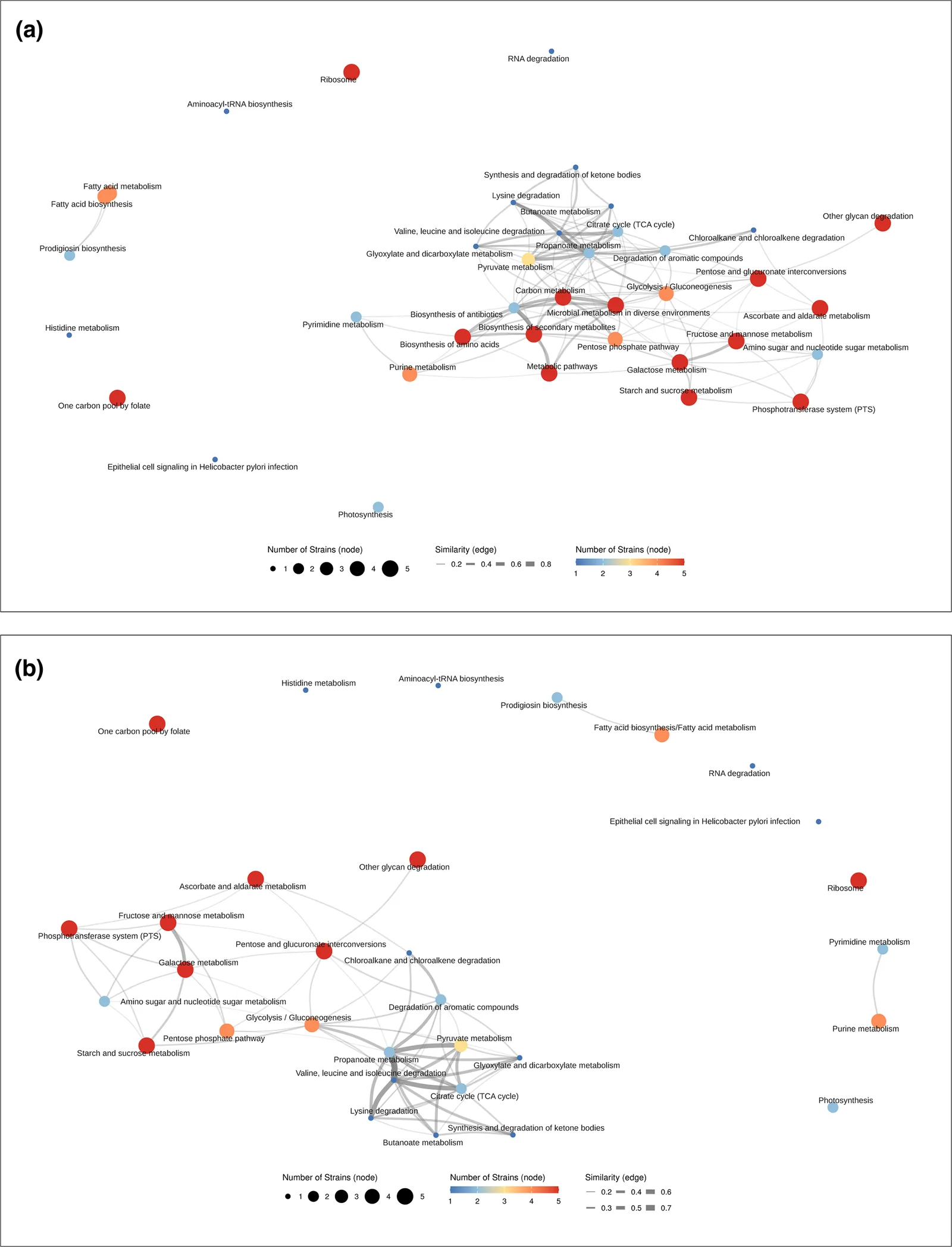
Networks of enriched pathways in *S. pyogenes* exposed to human serum (exported from our Shiny app). The networks were constructed from the outputs of DE analysis (**a, b**). Network filtering was applied to reduce visual clutter from the dense network (**b**). Nodes represent pathways, and connected edges represent significant pairwise similarity between enriched functional annotations or pathways calculated using Jaccard’s similarity score. The edge lengths and widths reflect pathway similarity. Node sizes and colours represent the number of strains sharing the same enriched pathways. Alternatively, edge width can be used to present the number of strains with interaction between pathways, as selected by the user.

The network highlights major pathways supporting *S. pyogenes* adaptation to serum, including carbon metabolism, ribosomal function and fatty acid metabolism. The central metabolic hub, grouped by shared biological molecules, contains highly interconnected pathways involved in the metabolism of carbohydrates and saccharides (fructose and mannose metabolism, galactose metabolism), aas and nts. This case study uncovered substantial reprogramming of carbon metabolism in *S. pyogenes* when exposed to serum. Several interconnected pathways showed coordinated shifts, reflecting the bacterium’s metabolic adaptation to the host environment. Studies have emphasized the importance of specific carbon flow patterns that support streptococcal fitness during infection [36, 37]. The reprogramming of carbon flow has been suggested as a potential therapeutic target [37]. Overall, our analysis indicates that host nutrient scavenging is prioritized over biosynthesis and that fermentation is upregulated in *S. pyogenes* to support energy expenditure and defence mechanisms.

## Discussion

Rapid advances in high-throughput omics technologies have enabled researchers to capture multilayered and systemic information from a single sample collection, which cannot be resolved by single omic analyses, highlighting the need for integrative multi-omics approaches [38]. The key challenge in multi-omics research is increasingly shifting from data generation to effective downstream analysis, such as data integration, functional interpretation and biological contextualization [39–42]. Building on established tools and introducing no new algorithm, we propose a flexible and well-documented analytical framework for microbial multi-omics data integration and visualization to facilitate biological interpretation.

Using an existing *S. pyogenes* multi-omics dataset [1], the structure of the workflow is generalizable to a wide range of experimental designs and microbial systems. Focused on downstream data integration, the workflow supports both knowledge-based and data-driven hypothesis formulation, and simplifies the use of powerful multivariate and network-based methods for biomarker discovery and pathway-level interpretation within microbial datasets. The combined use of a multi-block (s)PSL-DA framework and network integration of pathway enrichment outputs allows for the systematic consideration of multiple biological layers, thereby revealing key molecules and pathways resulting from experimental conditions. The network-based analyses applied in our case study captured both shared and strain-specific responses, demonstrating the approach’s capacity to disentangle conserved processes from context-dependent adaptations.

The Shiny app delivers interactive networks and a ranking and filtering system, allowing users to prioritize the most informative and discriminatory pathways across conditions in real time. It is designed to filter out overly broad categories, e.g. general GO terms such as ‘metabolic process (GO:0008152)’, which can obscure meaningful insights. By instead focusing on specific GO terms, e.g. ‘glycolytic process (GO:0006096)’ and ‘pentose-phosphate pathway (GO:0006098)’, the approach enables more precise and interpretable results in microbial systems. The node prioritization function in the Shiny app directly facilitates the identification of targeted pathways (e.g. conserved or strain-specific pathways) and inter-pathway relationships.

While our workflow provides a systematic framework for microbial multi-omics integration, inherent limitations should be acknowledged. In the data preprocessing step, consideration must be given to the selection of appropriate quality control (QC) tools and normalization strategies based on the omics types and experimental design. This critical step can substantially influence all downstream analyses within the workflow. In the DIABLO framework, integration of unequal sample sizes and instability of supervised feature selection should be acknowledged. While the DIABLO model can handle missing data for prediction on new samples, training the model on datasets with unequal sample sizes remains challenging. This limitation arises from the underlying statistical framework, Generalized Canonical Correlation Analysis, which relies on maximizing the covariance between latent components across different omics blocks. Since covariance estimation requires paired observations, unequal sample sizes across omics blocks (e.g. 100 transcriptomic samples but only 80 metabolomic samples) hinder the direct computation of inter-block relationships during model training. Additionally, unbalanced phenotypic group sizes may introduce bias in error rate estimation. The BER can be used during cross-validation to guide component selection and reduce the influence of class imbalance. In studies with small sample sizes, supervised feature selection can be unstable, especially during sample partitions in the cross-validation step. To address this, a feature stability score, reflecting the frequency of selection across resampling iterations, should be incorporated into the biological interpretation. For gene annotation, the functional interpretation relies heavily on the availability and accuracy of selected databases (e.g. KEGG and GO). In microbial systems, many uncharacterized genes may be excluded from downstream pathway analysis. In PEA, well-studied genes are more likely to appear in pathway enrichment results. Although standard FDR correction helps mitigate false positives, users should be aware of this bias.

Looking forward, we anticipate that multi-omics technologies will become routinely used by scientists to understand microbial behaviour within complex biological contexts. The workflow presented here supports researchers by integrating multi-omics data and translating these into biological insights and testable hypotheses in systems microbiology.

## Supplementary material

10.1099/mgen.0.001860 Supplementary Material 1.

## Abbreviations

BER: balanced error rate
DE: differential expression
DIABLO: Data Integration Analysis for Biomarker discovery using Latent variable approaches for Omics studies
GSEA: Gene Set Enrichment Analysis
logFC: log fold change
NIPALS: non-linear iterative PLS
ORA: over-representation analysis
PCA: principal component analysis
PEA: pathway enrichment analysis
PLS-DA: partial least squares discriminant analysis
(s)PLS-DA: (sparse) PLS-DA
