# Supplementary material for: A practical resource for multi-omics data integration in microbial systems
Source: Microb Genom. 2026 Nov 4;12(11):001860. doi: 10.1099/mgen.0.001860 (PMC13733548; doi:10.1099/mgen.0.001860)
Supplement: Supplementary file 1 [file mgen-12-01860-s001.pdf]

Supplementary Figures and Tables

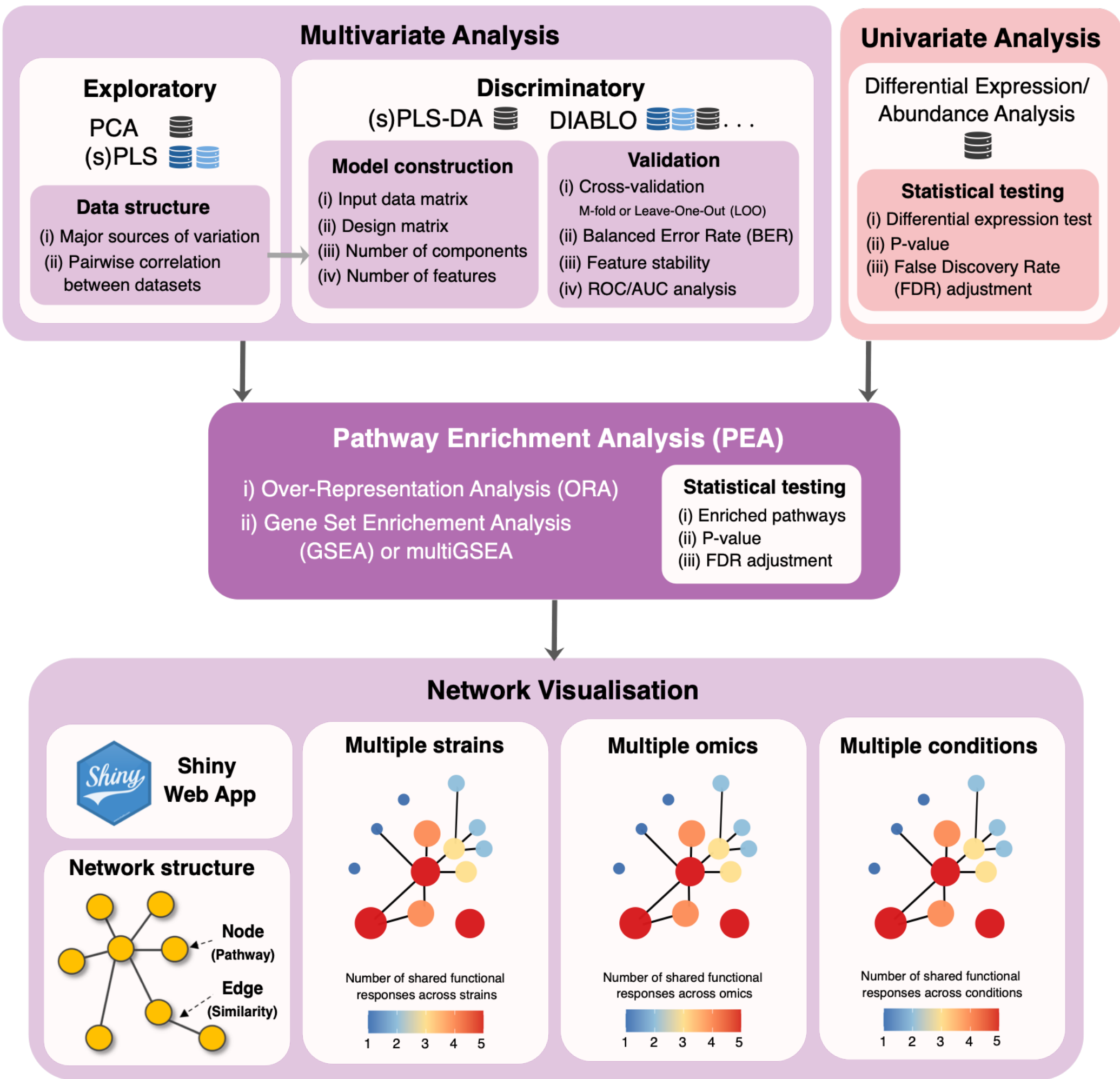

Figure S1. Detailed computational workflow for multi-omics data integration, supporting user decisions and parameter tuning.

The core elements of the workflow are highlighted in purple. Both multivariate and univariate analyses are presented in the initial step of this workflow. In this context, multivariate analysis consists of two main parts, covering exploratory (e.g., PCA and (s)PLS) and discriminatory (e.g., (s)PLS-DA and DIABLO) analyses. PCA and PLS methods facilitate exploration of global data structure and relationships across omics datasets which support users' decision-making on model construction in PLS-DA or DIABLO framework (e.g. design matrix). DIABLO facilitates supervised integration and classification. This framework enables selection of correlated features across datasets and highlights discriminative signatures linked to experimental conditions. The main components for model construction include (i) input data matrix, (ii) design matrix, (iii) Number of components, and (iv) number of features. More detailed information on DIABLO model design and parameter tuning is in the **Supplementary Methods**. In addition, validation plays an important role during model construction. The optimal number of components and features can be estimated by cross-validation (e.g., M-fold and leave-one-out (loo)) where classification performances are accessed. In general, M-fold

cross-validation is recommended, except when the number of samples is very small. In addition, Balanced Error Rate (BER) is commonly used to evaluate the model performance during the parameter-tuning step. The selected features represent the most discriminative and highly correlated molecular signatures across the multi-omics dataset, as determined by minimisation of the balanced error rate. The final model performance can be evaluated using ROC-based metrics to assess classification performance. For biological interpretation, the loading values derived from the DIABLO model reflect the importance of each feature in driving the separation. Features with larger absolute loading values are the key contributors to the observed differences between conditions. In studies with small sample sizes, supervised feature selection can be unstable, especially during sample partitions in the cross-validation step. Optionally, a feature stability score, which reflects the frequency of selection across resampling iterations, can be additionally considered during the biological interpretation.

The second part of the workflow contains pathway enrichment and network analysis. Pathway enrichment analysis (PEA) through the *clusterProfiler* package supports both over-representation analysis (ORA) and gene set enrichment analysis (GSEA). Optionally, a GSEA-based pathway enrichment analysis for multi-omics data (multiGSEA) can be considered in pathway enrichment measures. In this workflow, results (e.g. p-value and adjusted p-value (False Discovery Rate (FDR) correction) from statistical testing (e.g. Benjamini-Hochberg correction) are used to identify enriched pathways. An adjusted p-value threshold of < 0.05 is commonly used for statistical significance, whereas a p-value threshold of < 0.05 can be used for exploratory purposes. To support interactive exploration of enrichment pathway networks, we developed a dedicated R Shiny web application called Network of Enriched Pathways (https://warasinee.shinyapps.io/Network_App/). More comprehensive information is in the **Interactive Network Visualisation via a Shiny Web Application** section.

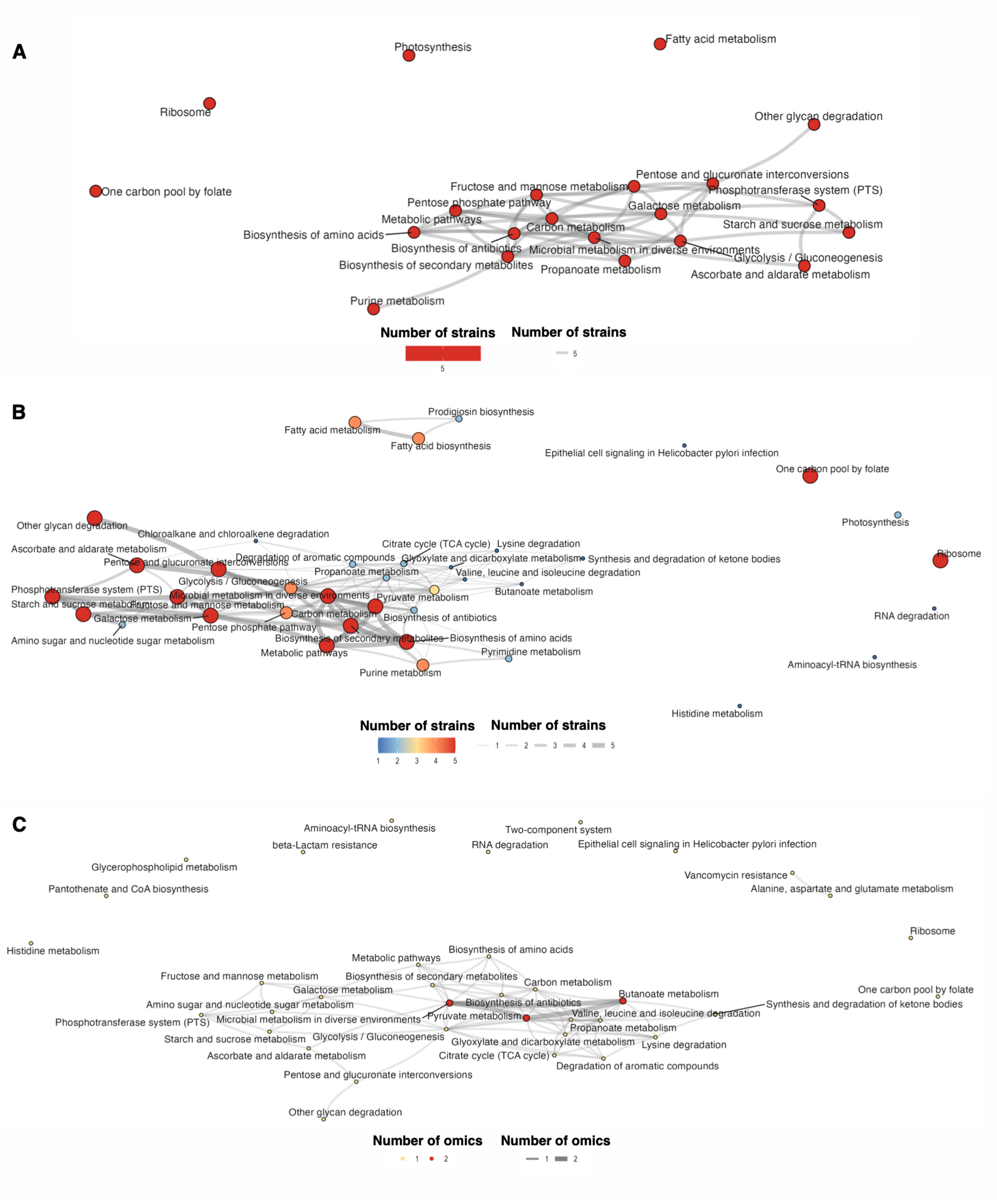

Figure S2. Networks of enriched pathways in *Streptococcus pyogenes* exposed to human serum. The networks were constructed from the outputs of multi-block PLS-DA (A) and DE analysis (B-C). Nodes represent pathways, and connected edges represent significant pairwise similarity between enriched functional annotations or pathways calculated using Jaccard's similarity score. Node sizes and colours represent the number of strains (A-B) or omics (C) sharing the same enriched pathways. Edge widths indicate the number of strains or omics with interaction between pathways.

All pathway enrichment results (ORA and GSEA) were generated via the *clusterProfiler* package. In this case study, an adjusted p-value (from FDR correction using the Benjamini-Hochberg method) threshold of < 0.05 was used for statistical significance, whereas a p-value threshold of < 0.05 was used for exploratory purposes (i.e. applied to proteomic data in **Figure S2C**).

Pathway-level networks were generated from three complementary analyses: (i) enrichment of discriminant features identified by multi-block PLS-DA, highlighting shared serum responses across strains (**Figure S2A**); (ii) pathway enrichment from single-omics differential expression analyses, performed using GSEA with the KEGG database, showing both shared and strain-specific responses (**Figure S2B**); and **(**iii) integration of responses across multiple omics layers (i.e. transcriptomic and proteomic layers) within a single strain (SP444) (**Figure S2C**). These networks integrate diverse signals into a coherent systems-level view, allowing researchers to identify and prioritise key pathway functions and molecules while providing a foundation for generating testable hypotheses. The first two networks highlight major pathways supporting *S. pyogenes* adaptation to serum, including carbon metabolism, ribosomal function, and fatty acid metabolism. The central metabolic hub, grouped by shared biological molecules, contains highly interconnected pathways involved in the metabolism of carbohydrates and saccharides, amino acids, and nucleotides. Notably, downregulation of fatty acid metabolism at the transcript level was observed across four strains except strain SP444 (**Figure S2B-S2C**, **Table S4**). In SP444 exposed to serum, metabolic shifts toward amino acid and alternative carbon usage are indicated by significantly upregulated pathways (e.g. butanoate metabolism, lysine degradation, valine, leucine, isoleucine degradation, and glyoxylate and dicarboxylate metabolism).

Table S1. List of variables identified as key drivers of *S. pyogenes* adaptation to human serum

Dataset	Selected Variable	Annotation	Loading value
Transcriptome	g012884	acid phosphatase AphA	0.197
Transcriptome	g001781	30S ribosomal protein S21	-0.021
Transcriptome	g009526	DUF3114 domain-containing protein	-0.048
Transcriptome	g011628	ketoacyl-ACP synthase III	-0.055
Transcriptome	g007017	pantetheine-phosphate adenylyltransferase	-0.071
Transcriptome	g001918	pyridoxal phosphate-dependent aminotransferase	-0.079
Transcriptome	g001273	pyroglutamyl-peptidase I	-0.092
Transcriptome	g002984	hypoxanthine phosphoribosyltransferase	-0.112
Transcriptome	g012223	DegV family protein	-0.12
Transcriptome	g002326	aspartate--ammonia ligase	-0.148
Transcriptome	g004005	50S ribosomal protein L28	-0.17
Transcriptome	g006376	amino acid ABC transporter ATP-binding protein	-0.204
Transcriptome	g008738	redoxin NrdH	-0.242
Transcriptome	g007331	MarR family transcriptional regulator	-0.255
Transcriptome	g011866	DUF979 domain-containing protein	-0.261
Transcriptome	g009109	50S ribosomal protein L32	-0.263
Transcriptome	g012346	amino acid ABC transporter substrate-binding protein	-0.337
Transcriptome	g006129	DUF969 domain-containing protein	-0.356
Transcriptome	g012489	ParB/RepB/Spo0J family partition protein	-0.384
Transcriptome	g012562	enoyl-CoA hydratase	-0.409
Proteome	g011169	SEC10/PgrA surface exclusion domain-containing protein	0.491
Proteome	g011237	formate C-acetyltransferase	0.448
Proteome	g005460	aspartate carbamoyltransferase	0.23
Proteome	g002782	orotate phosphoribosyltransferase	0.183
Proteome	g009654	L-lactate oxidase	0.071

Dataset	Selected Variable	Annotation	Loading value
Proteome	g012346	amino acid ABC transporter substrate-binding protein	-0.011
Proteome	g000992	glutamine-hydrolyzing GMP synthase	-0.02
Proteome	g000951	ATP-dependent zinc metalloprotease FtsH	-0.034
Proteome	g008162	YbaB/EbfC family nucleoid-associated protein	-0.037
Proteome	g006535	signal peptidase I	-0.041
Proteome	g012538	hypothetical protein	-0.052
Proteome	g009368	efflux RND transporter periplasmic adaptor subunit	-0.094
Proteome	g011244	branched-chain amino acid aminotransferase	-0.141
Proteome	g005099	bifunctional acetaldehyde-CoA/alcohol dehydrogenase	-0.143
Proteome	g000226	tyrosine--tRNA ligase	-0.18
Proteome	g010045	LTA synthase family protein	-0.202
Proteome	g011158	cation-translocating P-type ATPase	-0.212
Proteome	g011800	ACP S-malonyltransferase	-0.217
Proteome	g010545	ABC transporter permease	-0.238
Proteome	g006376	amino acid ABC transporter ATP-binding protein	-0.433
Metabolome	METAB_HMDB0000067	Cholesterol	0.465
Metabolome	METAB_HMDB0000056	Beta-Alanine	0.293
Metabolome	METAB_HMDB0003424	1-Hexadecanol	0.252
Metabolome	METAB_HMDB0000182	L-Lysine	0.246
Metabolome	METAB_HMDB0000243	Pyruvic acid	-0.02
Metabolome	METAB_HMDB0000262	Thymine	-0.268
Metabolome	METAB_HMDB0000210	Pantothenic acid	-0.308
Metabolome	METAB_HMDB0000362	2-Phosphoglyceric acid	-0.326
Metabolome	METAB_HMDB0000807	3-Phosphoglyceric acid	-0.354
Metabolome	METAB_HMDB0000073	Dopamine	-0.419

Table S2: Computational Resource Requirements

Script	Analytical Module	Key Computationally Intensive Step	Total Runtime	Peak Memory
1. PCA.Rmd	Exploratory analysis/PCA	Model fitting	~4 sec	199.7 MB
2. PLS.Rmd	Exploratory analysis/Pairwise PLS	Model fitting	~60 sec	222.1 MB
3. DIABLO.Rmd	Discriminatory analysis/Integrative modeling	Tuning step	~170 sec (~3 min)	243.2 MB
4.ORA-GSEA.Rmd	Pathway Enrichment Analysis (PEA)	GSEA permutations	~827 sec (~14 min)	907.2 MB
5. Node-edge.Rmd	Network data preparation	Pathway database loading	~7 sec	460.9 MB
6. Network.Rmd	Network analysis	Network construction	~2.6 sec	112.7 MB

Table S3: Workflow Inputs, Outputs, and Dependencies

Script	Module	Input	Input Format	Key R packages	Output	Output Format
1. PCA.Rmd	Exploratory analysis/PCA	Omics data matrices	.csv/.rds	mixOmics	Loading plot, Individual/Sample plot	.pdf
2. PLS.Rmd	Exploratory analysis/Pairwise PLS	Omics data matrices	.csv/.rds	mixOmics	Correlation plot, inferred design matrix values	.pdf
3. DIABLO.Rmd	Discriminatory analysis/Integrative modeling	Omics data matrices, sample metadata, design matrix	.csv/.rds	mixOmics	Tuned DIABLO model, selected feature, stability scores, Loading plot, Sample plot, ROC plot	.rds/.csv/.pdf
4.ORA-GSEA.Rmd	Pathway Enrichment Analysis (PEA)	(i) DEG from Differential Expression/Abundance (DE) analysis<br><br>(ii) Selected feature lists from DIABLO	.csv/.rds	clusterProfiler	Enriched pathway results (ORA & GSEA)	.rds
5. Node-edge	Network data preparation	Enriched pathway results	.rds	clusterProfiler, enrichplot	Node and edge tables	.tsv
6. Network.Rmd	Network analysis	Node and edge tables	.tsv	igraph, ggraph, tidygraph	Network plot, similarity matrix	.tsv/.pdf

Table S4: Pathway Enrichment Results (related to Figure 6)

Strain	Description	setSize	enrichmentScore	NES	pvalue	p.adjust	qvalue
5448	Fatty acid biosynthesis	24	-0.68	-2.20	9.19E-06	5.42E-04	4.28E-04
HKU419	Fatty acid biosynthesis	24	-0.69	-2.14	1.81E-05	1.02E-03	7.77E-04
PS003	Fatty acid biosynthesis	23	-0.72	-2.47	1.00E-06	2.45E-05	1.81E-05
PS006	Fatty acid biosynthesis	23	-0.61	-2.05	4.23E-04	4.42E-03	3.37E-03
SP444	Fatty acid biosynthesis	23	-0.28	-0.92	5.69E-01	7.41E-01	5.00E-01
5448	Fatty acid metabolism	29	-0.62	-2.12	4.64E-05	1.18E-03	9.33E-04
HKU419	Fatty acid metabolism	29	-0.65	-2.11	2.31E-05	1.05E-03	8.03E-04
PS003	Fatty acid metabolism	27	-0.65	-2.33	1.03E-05	1.72E-04	1.27E-04
PS006	Fatty acid metabolism	28	-0.57	-2.01	6.38E-04	6.14E-03	4.68E-03
SP444	Fatty acid metabolism	27	0.27	0.91	6.03E-01	7.66E-01	5.17E-01
5448	Butanoate metabolism	36	0.38	1.38	7.12E-02	2.46E-01	1.94E-01
HKU419	Butanoate metabolism	36	0.34	1.20	1.92E-01	4.15E-01	3.16E-01
PS003	Butanoate metabolism	36	0.33	1.09	3.37E-01	5.54E-01	4.10E-01
PS006	Butanoate metabolism	36	0.32	1.12	2.83E-01	5.20E-01	3.96E-01
SP444	Butanoate metabolism	36	0.48	1.75	3.82E-03	2.24E-02	1.51E-02
5448	Lysine degradation	23	-0.23	-0.74	8.46E-01	9.18E-01	7.24E-01
HKU419	Lysine degradation	23	0.33	1.07	3.56E-01	5.66E-01	4.30E-01
PS003	Lysine degradation	24	0.37	1.13	3.00E-01	5.12E-01	3.79E-01
PS006	Lysine degradation	23	0.32	0.99	4.66E-01	6.79E-01	5.17E-01
SP444	Lysine degradation	24	0.53	1.76	5.97E-03	3.01E-02	2.03E-02
5448	Valine, leucine and isoleucine degradation	39	-0.32	-1.17	2.21E-01	4.69E-01	3.70E-01
HKU419	Valine, leucine and isoleucine degradation	39	0.34	1.23	1.57E-01	3.74E-01	2.84E-01
PS003	Valine, leucine and isoleucine degradation	40	-0.30	-1.17	1.92E-01	3.87E-01	2.87E-01
PS006	Valine, leucine and isoleucine degradation	40	-0.31	-1.21	1.65E-01	3.87E-01	2.95E-01
SP444	Valine, leucine and isoleucine degradation	40	0.51	1.91	6.47E-04	5.21E-03	3.52E-03
5448	Glyoxylate and dicarboxylate metabolism	55	0.28	1.12	2.63E-01	5.11E-01	4.03E-01
HKU419	Glyoxylate and dicarboxylate metabolism	55	0.37	1.45	2.84E-02	1.17E-01	8.90E-02
PS003	Glyoxylate and dicarboxylate metabolism	53	0.30	1.05	3.94E-01	6.11E-01	4.52E-01
PS006	Glyoxylate and dicarboxylate metabolism	54	0.30	1.12	2.76E-01	5.14E-01	3.91E-01
SP444	Glyoxylate and dicarboxylate metabolism	54	0.41	1.65	5.53E-03	2.91E-02	1.97E-02

Supplementary Methods

Computational Implementation and Reproducibility

All analyses were performed in R (version 4.2.3, 2023-03-15) on an Apple Silicon Mac (aarch64-apple-darwin20, macOS 26.5.1) across six R Markdown scripts, each corresponding to a distinct analytical module. All R packages used are available on CRAN and Bioconductor and are expected to be cross-platform compatible. Details of all installed R packages and their resources from the R environment used in the workflow are stored in a YAML file (called yamlpack.yml) on GitHub, which can be restored via the *yamlpack* package.

The input data comprised three omics layers, including metabolome (60 samples × 129 features), proteome (60 samples × 993 features), and transcriptome (60 samples × 2,239 features), totalling 3,361 molecular features across 60 samples. **Table S2** summarises the runtime and memory requirements for each script. Overall, the most computationally demanding steps were GSEA permutation testing and DIABLO model tuning via cross-validation, while data preprocessing, exploratory analysis, and network visualisation steps were completed in under 10 seconds. Peak memory usage reached 907 MB during pathway enrichment analysis, driven primarily by pathway database loading and GSEA permutation-based null distribution estimation. All remaining analytical steps required less than 250 MB. The complete end-to-end pipeline is completed in approximately 18 minutes and can be executed on a standard laptop without high-performance computing resources, though a minimum of 8 GB RAM is recommended to accommodate pathway database loading comfortably.

Considerations for Model Design and Parameter Tuning

Generally, users are expected to perform data-driven tuning for optimal results. There is no "default" number of components and features, as the optimal number depends on the specific biological context where they are selected based on the best predictive performance.

While K-1 components are commonly recommended (where K is the number of phenotype groups), an arbitrarily high number of components is used in generating the initial DIABLO model for the tuning step.

For feature selection, users are expected to provide a grid of values for the tune function, where a small number of variables (<50 with steps of 5 or 10) is often sufficient for classification performance.

To construct the design matrix, a matrix can be specified between 0 and 1 to represent relationship structure between two given dataframes, thereby controlling trade-off between identifying correlated features and maximising group discrimination. While the model with null design (design matrix = 0) focuses on discrimination, full design (design matrix = 1) aims to identify a common source of variation (or correlated information) shared across all omics layers. A moderately weighted design (design matrix = 0.5) represents a middle ground between the two modelling strategies, capturing a significant amount of shared information without affecting group separation. The diagonal is set to zero so that the relationship of a dataset to itself is not considered.

Notably, multivariate supervised methods such as PLS-DA and DIABLO require cross-validation to mitigate the risk of overfitting and ensure unbiased classification results, especially in studies with small sample sizes where the number of features exceeds the number of samples.
